# Development of coordination and muscular fitness in children and adolescents with parent-reported ADHD in the German longitudinal MoMo Study

**DOI:** 10.1038/s41598-022-06139-1

**Published:** 2022-02-08

**Authors:** Elke Opper, Olga Kunina-Habenicht, Doris Oriwol, Anke Hanssen-Doose, Janina Krell-Roesch, Robert Schlack, Annette Worth, Alexander Woll

**Affiliations:** 1 University of Education Karlsruhe, Bismarckstr, 10, 76133 Karlsruhe, Germany; 2 Department of Rehabilitation Sciences, T U Dortmund University, Dortmund, Germany; 3 Institute of Sports and Sports Science, Karlsruhe Institute of Technology, Karlsruhe, Germany; 4 Department of Epidemiology and Health Reporting, Robert Koch Institute, Berlin, Germany

**Keywords:** ADHD, Epidemiology, Paediatric research

## Abstract

This study examined the development of muscular fitness and coordination in children and adolescents with and without attention deficit hyperactivity disorder (ADHD) over a period of 11 years. Data was collected in three measurement waves as part of the longitudinal, representative Motorik-Modul (MoMo) study in Germany (2003–2006, 2009–2012, 2014–2017). The overall sample comprised 2988 participants (253 with ADHD, 65% males; 2735 non-ADHD, 47% males; mean age 9 years). Structural equation modeling was conducted, and the estimated models had a good fit. No differences in muscular fitness were observed between participants with and without ADHD. Participants with ADHD had a lower coordinative performance at first measurement than those without ADHD. The difference in coordinative performance persisted throughout the study period.

## Introduction

Attention deficit hyperactivity disorder (ADHD) is among the most commonly diagnosed mental disorders in children and adolescents. ADHD has a prevalence rate of approximately 5.3% worldwide which has remained stable in recent years^1–3^. In the second wave of the nationally representative German National Health Examination and Interview Survey for children and adolescents KiGGS^4^, the prevalence of parent-reported ADHD diagnosed by a physician or psychologist at some point in the children’s or adolescents’ lives is somewhat lower, i.e., 4.4% in 3- to 17-year-old individuals^5^. Significantly more boys (6.5%) than girls (2.3%) in Germany are affected by ADHD, which is largely consistent with international findings even though diagnosis rates have recently risen more sharply in girls than in boys^6,7^.

ADHD describes a cross-situational developmental disorder that usually has its onset in childhood. Inattention, impulsivity (deficit in inhibitory control), and/ or motor agitation are considered core symptoms of ADHD. A diagnosis of ADHD is complex and requires the occurrence of symptoms over a period of at least 6 months in several areas of life (e.g., school, family, peer group). Although the diagnostic criteria have been updated several times, the clinical description of the core symptoms of ADHD has remained essentially unchanged over several decades^1,8^. ADHD is usually diagnosed once children enter school. In school, they are required to sit and maintain focus and concentration for extended periods of time, and are expected to only speak when called upon to do so by the teacher. This can be particularly challenging for children with ADHD who often present with motor restlessness or verbal disturbances in the classroom setting. Furthermore, they often cannot sufficiently profit from learning conditions and methods anchored in school and club structures^9^. In addition, children and adolescents with ADHD are particularly affected by the adverse consequences of the changing environmental situation, such as increasing digitalization and a related increase in sitting hours, a reduction in opportunities for free movement and play, and increasingly built-up open spaces. To this end, Roth^9^ points out that children and adolescents with ADHD require far more physical activity time and spaces than their peers without ADHD. Also, a recent research derived from three waves of the KiGGS study showed that physical activity was partly associated with lower specific symptoms of hyperactivity and inattention, as was general mental health^10^. Having a parent-reported ADHD diagnosis, however, did not interact with physical activity with respect to general mental health. Therefore, the authors concluded that the association between physical activity and mental health problems may not be influenced by the diagnosis of ADHD^10^.

About 60 to 80% of individuals with ADHD have comorbidities in psychological, physical, social as well as motor domains^11^, and about 75% of affected children and adolescents develop a comorbid disorder^1,8^. Mahone^12^ thus emphasizes the relevance of assessing motor developmental status as an important component in the diagnosis of ADHD. Indeed, research has shown that children and adolescents with ADHD have motor deficits^13^, particularly with regard to coordination and balance performance (e.g.,^14–19^). Albeit few studies reported no difference in motor performance between children with versus without ADHD^20^ or reported that ADHD subjects had better motor performance than non-ADHD subjects^21^.

However, most previous studies are limited by cross-sectional designs, and are only comparable to a limited extent due to differences in methodological approaches, including large variety in sample sizes or lack of comparison or control groups. ADHD does not necessarily remit during adolescence but persists into adulthood in approximately 30% of affected persons, indicating the chronic nature of this disorder^22^. It is therefore critical to also examine potential long-term motor deficits and changes in motor performance. This is preferably done by conducting longitudinal studies among children with ADHD compared to those without^23^. To date, there is a lack of longitudinal, representative studies that examined motor performance trajectories among children and adolescents with ADHD, and that also compared motor performance among children and adolescents with versus without ADHD. In addition, available longitudinal research has mainly focused on cardiorespiratory fitness, e.g. a study derived from the European Youth Heart Study found that children with low cardiorespiratory fitness at the age of 9 years had a more than two-fold increased risk of increased ADHD symptoms at 6 years follow-up^24^.

To address this gap, our aim was to investigate based on the longitudinal, representative Motorik-Modul (MoMo) study, which is the in-depth motor module of the long-term, nationally representative German Health Interview and Examination Survey (KiGGS study)^4^, whether there are differences in motor performance, i.e. muscular fitness and coordination, between children and adolescents with and without parent-reported ADHD at baseline, and to compare the longitudinal development of motor performance between those with and without ADHD over a period of 11 years.

## Results

### Descriptive statistics

A description of the characteristics of the study samples is given in Table 1. The descriptive statistics (mean values of the percentiles, standard deviation and 95% confidence intervals) for all motor tests considered in the analyses (T1, measurement wave 2003–2006; T2, measurement wave 2009–2012; T3, measurement wave 2014–2017) are presented in Table 2, stratified by ADHD and non-ADHD participants. A percentile value of 1 reflects the lowest age- and sex-specific test performance, whereas a percentile of 99 reflects the best possible age- and sex-specific performance^25^. Overall, there is a trend for better test performance in all motor tests and across all three measurement points for non-ADHD as compared to ADHD subjects.

**Table 1:** Characteristics of the study samples (T1, T2, and T3).

Sample characteristics	Study sample T1 (2003–2006)	Study sample T2 (2009–2012)	Study sample T3 (2014–2017)	Overall T1–T3 (2003–2017)	
				ADHD	Non-ADHD
n (%)	2376	2821	2047	253 (9)	2735 (91)
Age; M ± SD	9.0 ± 3.8	14.9 ± 4.4	18.3 ± 4.5	9.4 ± 3.3	9.0 ± 3.6
Age; Min, Max	4.0, 17.9	4.2, 25.1	9.1, 31.7	4.0, 17.9	4.0, 17.9
Age; 95% CI	8.8–9.1	13.7–14.1	18.1–18.6	9.0–9.8	8.9–9.1
Male (%)|female (%)	48|52	48|52	46|54	65|35	47|53

Data are either the mean values (M) ± standard deviation (SD), minimum (Min), maximum (Max), and 95% CI = confidence interval or percent (%), age in column overall divided by ADHD/no ADHD is given for the subjects’ first measurement. *ADHD* attention deficit hyperactivity disorder.

**Table 2:** Descriptive results of motor performance and ADHD (T1, T2, and T3).

Motor performance	Study sample T1 (2003–2006)		Study sample T2 (2009–2012)		Study sample T3 (2014–2017)	
**Coordination**	ADHD	No ADHD	ADHD	No ADHD	ADHD	No ADHD
Jumping-sideways	28.0 ± 24.0 [24.5–31.4] N = 187	34.3 ± 26.5 [33.2–35.4] N = 2.155	45.6 ± 28.7 [41.8–49.3] N = 229	53.8 ± 28.4 [52.7–55.0] N = 2.456	45.4 ± 26.8 [40.9–50.0] N = 137	54.1 ± 28.0 [52.7–55.5] N = 1.581
Balancing backwards	37.7 ± 30.1 [33.4–42.1] N = 189	43.2 ± 30.3 [41.9–44.5] N = 2.175	46.4 ± 30.8 [42.4–50.4] N = 232	54.6 ± 29.9 [53.4–55.7] N = 2.474	43.1 ± 31.8 [37.8–48.5] N = 138	49.1 ± 30.9 [47.6–50.6] N = 1.581
Static stand	39.1 ± 31.1 [34.4–43.7] N = 187	44.3 ± 33.7 [42.8–45.7] N = 2.145	59.3 ± 36.9 [54.5–64.1] N = 232	70.4 ± 32.4 [69.1–71.7] N = 2.474	63.7 ± 36.5 [57.6–69.8] N = 139	70.9 ± 33.3 [69.2–72.5] N = 1.595
**Muscular fitness**	ADHD	No ADHD	ADHD	No ADHD	ADHD	No ADHD
Standing long jump	52.0 ± 30.5 [47.6–56.4] N = 187	54.2 ± 29.4 [53.0–55.4] N = 2.177	49.7 ± 29.1 [45.9–53.5] N = 229	53.6 ± 28.5 [52.5–54.7] N = 2.452	47.8 ± 30.5 [42.6–53.0] N = 137	50.3 ± 29.4 [48.9–51.8] N = 1.582
Push-ups	40.0 ± 28.4 [35.4–44.6] N = 152	47.8 ± 29.4 [46.3–49.2] N = 1.561	50.0 ± 29.5 [46.2–53.9] N = 225	52.3 ± 29.8 [51.1–53.5] N = 2.414	48.5 ± 32.0 [43.1–54.0] N = 137	52.3 ± 29.7 [50.8–53.7] N = 1.568
Sit ups	Not available in T1	Not available in T1	49.7 ± 29.8 [45.7–53.6] N = 225	53.3 ± 29.1 [52.1–54.5] N = 2.419	47.7 ± 29.0 [42.8–52.6] N = 138	47.3 ± 28.1 [48.9–48.7] N = 1.582

Data are the mean values (M) of the percentiles ± standard deviation (SD) and [95% CI = confidence interval]. A percentile value of 1 indicates the lowest possible percentile whereas a percentile value of 99 reflects the best possible percentile (Niessner et al.^25^). *ADHD* attention deficit hyperactivity disorder.

### Measurement models

**Figure 1:**
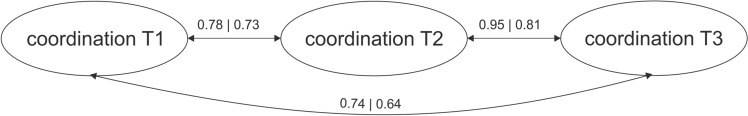
Simplified model for coordination (χ^2^ = 56.742; df = 39; *p* = 0.033; CFI = 0.996; RMSEA = 0.017). 1: 2003–2006; T2: 2009–2012; T3: 2014–2017. *df* degrees of freedom, *CFI* Comparative Fit Index, *RMSEA* root mean square error of approximation, *ADHD* attention deficit hyperactivity disorder. The first value before the vertical bar refers to the values in the ADHD group, while values after the vertical bar apply to the non-ADHD group. The presented results are based on the multi-group model with completely fixed factor loadings and intercepts over both groups as well as the restrictions over the measurement points according to the partial scalar measurement model over time.

**Figure 2:**
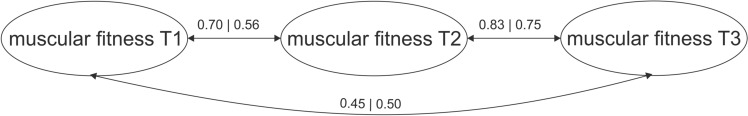
Simplified model for muscular fitness (χ^2^ = 41.316; df = 27; *p* = 0.038; CFI = 0.997; RMSEA = 0.019). T1: 2003–2006; T2: 2009–2012; T3: 2014–2017. *df* degrees of freedom, *CFI* Comparative Fit Index, *RMSEA* root mean square error of approximation, *ADHD* attention deficit hyperactivity disorder. The first value before the vertical bar refers to the values in the ADHD group, while values after the vertical bar apply to the non-ADHD group. The presented results are based on the multi-group model with completely fixed factor loadings and intercepts over both groups as well as the restrictions over the measurement points according to the partial scalar measurement model over time.

Figures 1 and 2 show the simplified measurement models for coordination and muscular fitness. All factor loadings were statistically significant and ranged from 0.47 to 0.83. The detailed measurement models, including the factor loadings and considered correlated errors, can be found in Supplementary Figs. 1 and 2.

The model fit of both measurement models was excellent (coordination: χ^2^ = 56.742; df = 39; *p* = 0.033; CFI = 0.996; RMSEA = 0.017; muscular fitness: χ^2^ = 41.316; df = 27; *p* = 0.038; CFI = 0.997; RMSEA = 0.019). There were high stabilities over time for coordination, represented by high latent correlations between the three measurement points (T1, T2, and T3), varying between 0.64 and 0.95. For coordination, all latent correlations were slightly higher in the ADHD group than in the non-ADHD group. For muscular fitness, the latent correlations were slightly lower than for coordination and ranged from 0.45 to 0.83. Two of three latent correlations for muscular fitness between the three measurement points were slightly higher in the ADHD group than in the non-ADHD group. However, the correlation between T1 and T3 was lower in the ADHD than in the non-ADHD group.

### Group comparisons over time

**Figure 3:**
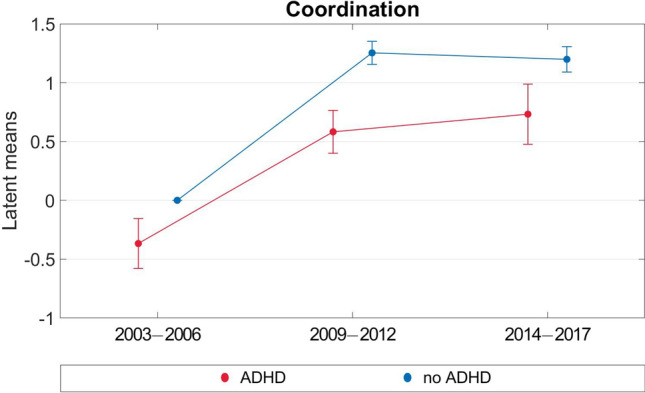
Multi-group analyses for development of coordination (2003–2017).

**Figure 4:**
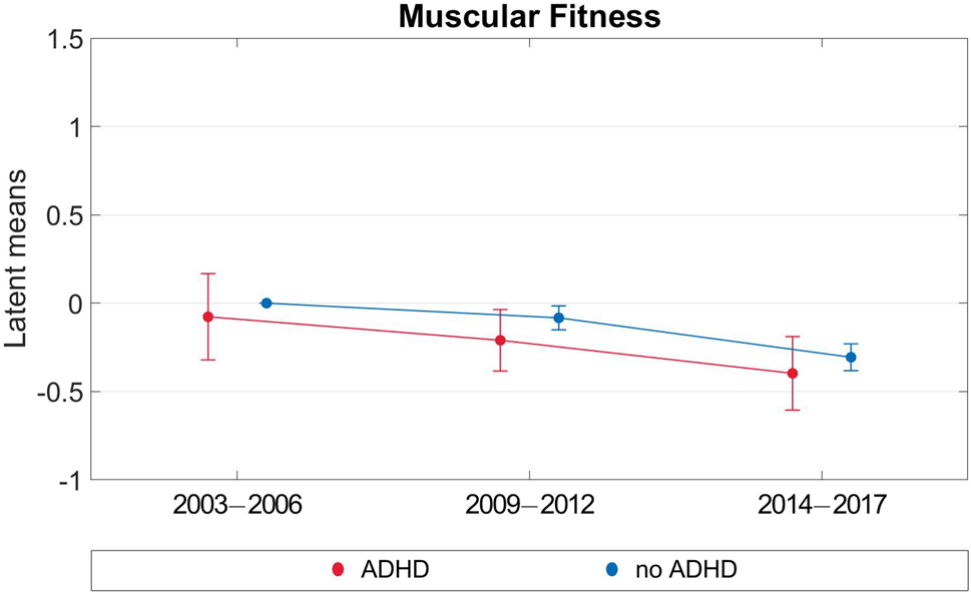
Multi-group analyses for development of muscular fitness (2003–2017).

The results of the multi-group analyses are reported in Table 3 and illustrated in Figs. 3 and 4. Coordination performance increased significantly for both the ADHD and non-ADHD groups from T1 to T2 and remained stable from T2 to T3. The ADHD group had a significantly lower level of coordination performance than the non-ADHD group at T1 and improved less from T1 to T2 than the non-ADHD group. The latent mean difference between T1 and T2 was approximately 0.95 SD for the ADHD group versus approximately 1.25 SD for the non-ADHD group, indicating a large effect for both. The latent mean differences between the ADHD and non-ADHD groups for coordination were 0.37 SD, 0.67 SD, and 0.47 SD at T1, T2, and T3, respectively.

**Table 3:** Latent means for non-ADHD group vs. ADHD group (based on the scalar multigroup model with partial scalar constrains over time).

Variable	Latent mean T1 (SE)	Latent mean T2 (SE)	Latent mean T3 (SE)
**Coordination**			
ADHD (N = 253)	− 0.367 (0.106)	0.582 (0.091)	0.732 (0.128)
Non-ADHD (N = 2735)	0*	1.253 (0.049)	1.198 (0.054)
**Muscular fitness**			
ADHD (N = 253)	− 0.077 (0.122)	− 0.210 (0.087)	− 0.397 (0.104)
Non-ADHD (N = 2735)	0*	− 0.082 (0.034)	− 0.306 (0.038)

*This value was constrained to 0 as a reference value for the estimation of all other latent means in the following time points as well as the latent means in the ADHD group. *ADHD* attention deficit hyperactivity disorder, *SE* standard error.

For muscular fitness there was a different pattern, i.e., the performance remained stable over time for both groups. The ADHD group had a slightly lower muscular fitness than the non-ADHD group at all three measurement points. However, the difference between the two groups appears less pronounced than that for coordination and should thus be interpreted with caution. The latent mean differences between ADHD and non-ADHD groups were 0.08 SD, 0.13 SD, and 0.09 SD in T1, T2, and T3, respectively.

## Discussion

For both ADHD and non-ADHD children and adolescents, there was high stability in coordination performance and muscular fitness over the study period of 11 years. These findings are in line with previous results of Schott^26^ and Blasquez Shigaki et al.^27^. In addition, we observed that the ADHD group had a lower level of coordination performance than the non-ADHD group at T1. Both groups improved their coordination performance over the study period, but the increase was greater in the non-ADHD group. With regard to muscular fitness, the two groups showed consistent development across all three measurement time points, and potential developmental differences were not observed.

These results are in line with previous studies that reported motor deficits in children with ADHD, particularly with regard to coordination and balance performance^14–17^. More than 30 years ago, Barkley^28^ reported a strong association between ADHD and deficits in motor control. Similarly, Shum and Pang^17^ identified deficits in balance, in association with impaired perception, in 43 elementary school children with ADHD compared to a control group of 50 children of the same age. In a review of motor developmental status, Hahn and Pieper^18^ concluded that children with ADHD have a reduced coordination performance, particularly in the areas of large motor, fine motor, and visual-motor skills. Fliers et al.^19^ conducted a cross-sectional study among 486 children with ADHD and 269 children free of ADHD symptoms aged between 5 and 19 years and assessed motor performance using questionnaires completed by parents (Developmental Coordination Disorder questionnaire, DCD) and teachers (Groningen Motor Observation scale). They observed coordination deficits in 34% of boys and 29% of girls with ADHD. The researchers also reported that both adolescents and younger children with ADHD were affected by coordination deficits, compared with healthy controls of the same age. In a follow-up study, Fliers et al.^14^ recruited 103 children (32 with ADHD, 18 unaffected siblings, 50 healthy controls) with a mean age of 10 years and objectively assessed motor performance using the Movement Assessment Battery for Children (MABC). In line with their previous findings, they observed a significantly poorer motor performance in ADHD participants compared to siblings and healthy controls (*p* = 0.038 and *p* < 0.001, respectively), with significant effects for all three MABC subscales (i.e., manual dexterity, balls skills, and balance). Overall, 63% of children with ADHD had motor deficits and 34% of the ADHD-group were classified in the clinical DCD-range^14,16^. Cho et al.^29^ also assessed motor function in 58 school-aged children with ADHD with a mean age of 9.6 years, and 70 children in the control group with a mean age of 9.2 years using the Bruininks–Oseretsky test of motor proficiency (second edition). They observed significantly lower mean standard scores on fine motor control (t = − 3.76, *p* < 0.001), manual coordination (t = − 6.87, *p* < 0.001), body coordination (t = − 7.14, *p* < 0.001), strength and agility (t = − 8.54, *p* < 0.01), and total motor composite score (t = − 9.32, *p* < 0.001) in children with ADHD than those in the control group. Similar findings were reported by Jeyanthi et al.^30^ who compared 16 Indian elementary school children aged 8–12 years with ADHD to 19 children without ADHD symptoms. Children with ADHD had significant deficits in motor performance and in gross and fine motor skills compared to those without ADHD symptoms. There was a significant difference in fine coordination (9-Hole Peg Test) between the two groups, i.e., children without ADHD symptoms completed the task faster (left hand: M = 22.03 s, SD = 2.20; right hand: M = 19.64 s, SD = 1.73) than children with ADHD (left hand: M = 51.65 s, SD = 4.27; right hand: M = 44.78 s, SD = 3.66), *p* < 0.001). In contrast, Colombo-Dougovito^21^ reported conflicting results based on a study of 51 elementary school-aged children (eight with ADHD; mean age = 7.75; 43 without ADHD, mean age = 8.23) that underwent seven predominantly endurance and strength-based tasks from "The President's Challenge" fitness program. Surprisingly, children with ADHD performed better in number of push-ups and curl-ups than their peers without ADHD (ADHD: curl-up, M = 31.56; push-up, M = 26.28; non-ADHD: curl-up, M = 27.62; push-up, M = 16.73). The inconsistent results may be explained by different methods used in assessing motor performance, in diagnosing ADHD, or by differences in samples, e.g. with regard to representativeness. Some authors, such as Colombo-Dougovito^21^ and Jeyanthi et al.^30^ also note their small sample sizes and call for larger-scale future studies on the relationship between motor performance and ADHD.

Overall, previous studies showed that children and adolescents with ADHD have motor deficits, particularly in coordination performance, compared to children and adolescents without ADHD; albeit conflicting results for example with regard to muscular strength were reported^21^. However, these findings were primarily derived from cross-sectional studies comparing the motor performance of children with and without ADHD.

Therefore, our longitudinal study builds on previous research by examining developmental trajectories in motor performance of children and adolescents with and without ADHD. Also, we used objective motor performance tests rather than questionnaire data, and also carried out repeated assessments of coordinative performance and muscular fitness in our study participants. Of note, we observed that parent-reported ADHD may serve as a predictor of a decline in coordinative performance that was not only statistically significant but also potentially clinically relevant (i.e., the difference in performance ranged between 10 and 20% depending on the test item). Since we observed differences in the developmental trajectories between ADHD and non-ADHD subjects in coordination but not muscular fitness, this may have important implications for the design and conduct of intervention studies in children and adolescents with ADHD, particularly those aiming at increasing motor performance. Also, future studies should focus on different dimensions of motor performance and aim at investigating potential mechanisms that may underlie the observed differences in motor performance developmental trajectories between children and adolescents with and without ADHD. Furthermore, future research should consider physical activity patterns of participants, e.g. activity type, frequency and intensity, as well as physical activity history and setting in which physical activity or sports are carried out.

The findings from our current study should be interpreted by considering its strengths and limitations. The major strengths of this longitudinal study are the population-based, representative sample, the long observation period from 2003 to 2017, and the objective and comprehensive measurement of coordination performance and muscular fitness. In addition, to address the research questions, this study estimated longitudinal SEMs that allow generalizing findings from single indicators or tasks to a higher construct level of coordination or muscular fitness. In addition, measurement invariance was explicitly tested as an important prerequisite for longitudinal analyses. One limitation of the study is that we did not consider physical activity behavior or sports participation in our analyses which may serve as moderators for the development of coordination and muscular fitness among children and adolescents with ADHD. Furthermore, as mentioned initially, ADHD does not necessarily remit over time^22^. Stability rates vary as a function of time and type of study (clinical vs. population-based) between 30 and 70%^31,32^. This corresponds well with the stability rates of the parent-reported diagnoses of child ADHD in the KiGGS study^33,34^. For example, the percentage of ADHD diagnoses that was consistently reported in the KiGGS cohort study 11 years after the baseline assessment was 37.3%^34^. An important assumption that we made to our analyses, however, is that ADHD is stable over the study period, which does not appear to be the case for all subjects. This may thus be considered a major limitation. However, in line with our hypothesis we observed that participants defined as ADHD subjects had poorer coordination performance over time than non-ADHD subjects although some of the former might have been misclassified. Given this, our results may need to be considered conservative. In addition, the observed ADHD prevalence of 9% in our study sample at baseline appears to be somewhat higher than what has previously been reported for the KiGGS study^5^. This is due to the fact that we have applied an extended ADHD definition, i.e., in our study, for case number reasons, we pooled participants with parent-reported ADHD diagnoses and the so-called “suspected cases” (SDQ hyperactivity subscale ≥ 7 and no parent report of an ADHD diagnosis) according to the case definition of Schlack et al.^5^. A statistical limitation of the reported analysis is that, with respect to measurement invariance over time, only partial scalar invariance over time was found for the two constructs of coordination and muscular fitness. This suggests that some indicators were not fully invariant over time. Furthermore, the results are representative for German children and adolescents, but may not be generalizable to other populations or children and adolescents from other countries.

In conclusion, our study provides additional evidence that motor skills of children and adolescents with ADHD develop over time, but remain below the performance levels of peers without ADHD. This underlines the need for increased promotion of physical activity in children and adolescents with ADHD. There is evidence that physical activity improves motor performance, social behavior and cognition, self-confidence, self-esteem, and social skills^35,36^. Children and adolescents with ADHD may also benefit from the positive effects of sports and physical activity on the motor, cognitive, and social development^37,38^, and there is a growing body of intervention studies examining the effects of physical activity on motor performance in children and adolescents with ADHD^35,36,39,40^. More longitudinal studies are required in the future that confirm our observations and examine the development of motor performance in children and adolescents with ADHD. It is also important to investigate the potential effects of physical activity interventions in this population and how they may differ from those in individuals without ADHD symptoms. The results of such studies would make it possible to provide children and adolescents with ADHD with even more targeted support regarding the development of critical motor skills.

## Methods

### Procedure

The presented analyses are based on data from the long-term, representative Motorik-Modul (MoMo) study in Germany. The MoMo study is a longitudinal study assessing motor performance and physical activity levels of children and adolescents in Germany. MoMo is an in-depth study of the KiGGS study^4^, the first representative health monitoring for children and youth in Germany conducted by the Robert Koch-Institute, Berlin. The KiGGS study was set up in 2003 and included a core survey, with MoMo as one of five modular in-depth studies carried out in KiGGS subsamples. The samples were selected randomly from official population registries in 167 sample points across Germany^41^. The children and adolescents first participated in KiGGS and afterward in MoMo. Three MoMo measurement waves have been conducted so far (T1: 2003–2006; T2: 2009–2012; and T3: 2014–2017). The MoMo participants and their parents were contacted individually and invited to test facilities nearby their homes in one of the 167 sample points, i.e., cities, municipalities^42,43^. In the test facilities, trained study staff accompanied and assisted the participants individually throughout the completion of physical tests and questionnaires. The MoMo study has a cohort sequence design with a cross-sectional and a longitudinal study arm. This article utilizes longitudinal data, i.e., participants from T1 were tested repeatedly at T2 and T3.

### Participants

The longitudinal MoMo sample includes a total of 2988 individuals who participated in at least two out of three measurement waves. 1268 individuals participated at all three measurement waves. 941 individuals participated at T1 and T2 but not at T3; 167 individuals participated at T1 and T3 but not at T2. As it was possible to enter the study at T2, there are 612 individuals who participated at T2 and T3 but not at T1. Overall, the sample for our analysis comprised 2376 participants at T1 (mean age 8.5 ± 3.7 years, missing data 612), 2821 participants at T2 (14.8 ± 3.8 years, missing data 167), and 2047 participants at T3 (20.0 ± 3.9 years, missing data 941). The mean age difference of the samples between T1 and T2 was 6.3 years, and that between T2 and T3 was 5.2 years. A description of the characteristics of the study samples is given in Table 1. The study was conducted according to the Declaration of Helsinki and was approved by the appropriate ethics committees (i.e., ethics committees of Charité Universitätsmedizin Berlin, the University of Konstanz, and the Karlsruhe Institute of Technology). The Federal Commissioner for data protection and freedom of information was informed about the study and approved it. Participation in the study was voluntary. The participants, parents, and custodians were informed about the aims and contents of the study, as well as about data protection, and gave their informed consent.

### ADHD (predictor variable)

In the KiGGS study, participants were classified as ADHD cases if their parents reported a lifetime ADHD diagnosis made by a physician or psychologist. Additionally, participants were considered cases with “suspected ADHD” if they scored ≥ 7 (“clinical range”) in the hyperactivity/ inattention subscale of the Strengths and Difficulties Questionnaire (SDQ)^33^. For the present analyses, both categories were pooled and defined as ADHD for case number reasons^5^. According to this definition, 253 (9%; 189 at T1, 64 at T2) participants were classified as ADHD subjects and 2735 (91%; 2187 at T1, 548 at T2) participants as non-ADHD subjects at baseline, i.e. their first measurement point. Of those participants with ADHD, 65% were males and 35% were females. Of non-ADHD subjects, 47% were males and 53% were females. There was no significant age difference between groups. Participants were aged between 4.0 and 17.9 years, the mean age of the ADHD group was 9.4 (± SD 3.3) years and that of the non-ADHD group was 9.0 (± SD 3.6) years.

### Motor performance—coordination and muscular fitness (outcome variables)

Motor performance was measured by a standardized test battery based on the systematization by Bös^25,44–46^. The battery includes tests from validated test batteries that have been used in research studies in the past^25,45,46^. For the analysis presented in this manuscript, we considered six out of twelve tests that cover the motor dimensions coordination and muscular fitness. All tests have been documented in a comprehensive manual^45^.

Coordination under time pressure was measured by the “jumping-sideways” test. In this test, the number of valid sideway jumps within 15 s carried out on a carpet mat (mean value of two repetitions) was assessed. Coordination under precision pressure was measured by the “balancing backwards” test. In this test, the participant is required to balance backwards on wooden beams of 3-m length and different widths: 3 cm, 4.5 cm, and 6 cm. A maximum score of eight steps on each beam without touching the floor is possible, and the sum of steps of two repetitions on each beam was recorded. Balance was measured by the “static stand” test which requires standing on the dominant leg on a wooden construction of 3-cm width and 7-cm height for one minute. The number of floor contacts of the contralateral leg within one minute during one test was recorded and there were no test repetitions^45,46^.

Muscular fitness was assessed by the “standing long jump”, “push-ups,” and “sit-ups” tests^45,46^. In the ‘standing long jump’ test, the maximum jump distance in cm from an upright standing position was recorded out of two jumps. In the ‘push-ups’ test, the number of complete push-ups completed within 40 s was recorded and there was no test repetition. In the ‘sit-ups’ test, the number of valid sit-ups completed within 40 s was assessed with no test repetition.

The dataset contained missing data, which can be attributed to either the study design or particularities of the variables as described below. The coverage of all variables at all three measurement points was considered good, i.e., 57.3–88.5% at T1, 88.3–90.6% at T2, and 57.1–58% at T3. Due to developmental reasons, the push-up and sit-up tests were only conducted in participants aged 6 years and older. Sit-ups were introduced in T2, thus there are no sit-up data available from T1.

Since age and sex differences were not the major interest of the current study, age- and sex-specific percentile curves were calculated for each of the six motor performance tests using the LMS transformation method^25^. According to this, a percentile value refers to the percentage of persons in the reference population with the same or lower performance. Thus, a percentile value of 1 represents the lowest possible performance, whereas a percentile value of 99 reflects the best possible performance^25^. We used percentile curves up to an age of 23 years for older participants. In cases where the age was above 23 years, the determined percentile in the last age range was assigned.

### Statistical analysis

Descriptive and correlational analyses were conducted using IBM SPSS Statistics for Windows, version 24 (IBM Corp., Armonk, N.Y., USA). A significance level of 0.05 was set as a threshold to determine statistical significance. As our research questions focus on inter-individual differences, longitudinal analyses were performed by applying a structural equation modeling (SEM) approach in Mplus, version 8.0^47^. The comparative fit index (CFI) and root mean square error of approximation (RMSEA) are reported as model fit indices, as well as χ^2^ values. According to Hu and Bentler^48^, RMSEA values ≤ 0.05 are considered as a good fit, and values between 0.05 and 0.08 are considered as an adequate fit. For the CFI, values of ≥ 0.90 reflect a satisfactory fit, whereas values ≥ 0.95 reflect an excellent fit. As a default option in Mplus, missing data were taken into account using the FIML approach.

As a requirement for the longitudinal analyses, we tested the measurement invariance for coordination and muscular fitness for both (a) ADHD vs. non-ADHD groups, and (b) the invariance over time. The results of the measurement invariance analysis are presented in Supplementary Tables 1 and 2. To ensure that the meaning of the constructs is comparable over groups and time, the scalar invariance level is required, i.e., factor loadings and intercepts are kept equal for groups or measurement points^49^. For the evaluation of model fit in the invariance analysis, we considered the traditional likelihood-ratio test, which is based on the chi-squared distribution. However, for large sample sizes, the likelihood-ratio test tends to flag statistical significance even if the observed deviations are not practically meaningful. Therefore, we also considered the criterion of CFI difference (ΔCFI) suggested by Cheung and Rensvold^50^ as it is less vulnerable to sample size. The authors suggested that “a value smaller than or equal to − 0.01 indicates that the null hypothesis of invariance should not be rejected”^50^. A certain level of measurement invariance was interpreted as established if one of the criteria, i.e., only the likelihood-ratio test, only the CFI difference criterion, or both, applied to the results.

In terms of invariance over groups (ADHD vs. non-ADHD group), full scalar invariance for the measurement models for coordination and muscular fitness could be established. Furthermore, with regard to the measurement invariance over time for both constructs, full metric invariance and partial scalar invariance over time could be established. For coordination and muscular fitness, we freed the intercept for the tasks “Balancing backwards” and “Push-ups within 40 s,” respectively. This indicates that these two single indicators were not completely invariant over time. The presented latent group comparisons are based on the multi-group models with completely fixed factor loadings and intercepts over both groups, as well as the restrictions over the measurement points according to the partial scalar measurement model over time.

In the latent multi-group model, the first measurement point in the non-ADHD group was used as a reference value and all other latent means were estimated in accordance with that reference. The differences between estimated latent means were interpreted similarly to the Cohen’s *d* effect size, i.e., a value of 0.2 indicates a group difference of 0.2 SD and represents a small effect; 0.5 indicates a medium effect, and d ≥ 0.8 indicates a large effect^51^. This statistical modeling approach allows us to model the covariance and the mean structure of the variables in both groups at the same time. It should be noted that high stability coefficients do not necessarily imply that there is no change in the latent means. In multi-group analysis, the regression coefficients refer to the rank order of persons in the sample. Thus, a regression coefficient of r = 1 between T1 and T2 indicates that the rank order of participants in the sample remains the same at T1 and T2. Nevertheless, it is still possible that all persons increase their abilities and show a higher latent mean at T2.

## Supplementary Information

Supplementary Figure 1.

Supplementary Figure 2.

Supplementary Table 1.

Supplementary Table 2.

## Data Availability

Data collected in the German Longitudinal MoMo Study are available on request from the corresponding author.
