# Supplementary material for: Development of coordination and muscular fitness in children and adolescents with parent-reported ADHD in the German longitudinal MoMo Study
Source: Sci Rep. 2022 Feb 8;12:2073. doi: 10.1038/s41598-022-06139-1 (PMC8827093; doi:10.1038/s41598-022-06139-1)
Supplement: Supplementary file 1 — Supplementary Figure 1. [file 41598_2022_6139_MOESM1_ESM.docx]

**Supplementary Figure 1**

*Detailed model for coordination* (χ² = 56.742; df = 39; *p* = 0.033; CFI = 0.996; RMSEA = 0.017).

*Note*. T1: 2003–2006; T2: 2009–2012; T3: 2014–2017 df: degrees of freedom; CFI: Comparative Fit Index; RMSEA: Root Mean Square Error of Approximation

The first value before the vertical bar refers to the values in the ADHD group, while values after the vertical bar apply to the no-ADHD group.

The presented results are based on the multi-group model with completely fixed factor loadings and intercepts over both groups as well as the restrictions over the measurement points according to the partial scalar measurement model over time.
