# Supplementary material for: Development of coordination and muscular fitness in children and adolescents with parent-reported ADHD in the German longitudinal MoMo Study
Source: Sci Rep. 2022 Feb 8;12:2073. doi: 10.1038/s41598-022-06139-1 (PMC8827093; doi:10.1038/s41598-022-06139-1)
Supplement: Supplementary file 3 — Supplementary Table 1. [file 41598_2022_6139_MOESM3_ESM.docx]

**Supplementary Table 1**

*Invariance testing for coordination and muscular fitness for ADHD (n = 253) vs. non-ADHD group (n =* *2,734) (without constrains over time)*

| Model | χ^2^ | df | *p* | CFI | RMSEA |
| --- | --- | --- | --- | --- | --- |
| **Coordination** | | |  |  |  |
| M1: Configural invariance ^a^ | 39.73 | 24 | 0.023 | 0.997 | 0.021 |
| M2: Metric invariance ^b^ | 46.22 | 30 | 0.030 | 0.996 | 0.019 |
| **M3: Scalar invariance** ^c^ | 54.65 | 36 | 0.024 | 0.996 | 0.019 |
| Difference between M1 and M2 | Δχ^2^ = 6.49, Δ*df =* 6, *p* = 0.37 | | | | |
| Difference between M2 and M3 | Δχ^2^ = 8.43, Δ*df =* 6, *p* = 0.21 | | | | |
| **Muscular fitness** | | |  |  |  |
| M1: Configural invariance ^a^ | 9.22 | 16 | 0.904 | 1 | 0 |
| M2: Metric invariance ^b^ | 20.89 | 21 | 0.466 | 1 | 0 |
| **M3: Scalar invariance** ^c^ | 30.82 | 26 | 0.235 | 0.999 | 0.011 |
| Difference between M1 and M2 | Δχ^2^ = 11.67, Δ*df =*5, *p* = 0.040 | | | | |
| Difference between M2 and M3 | Δχ^2^ = 9.93, Δ*df =* 5, *p* = 0.077 | | | | |

*Note.* CFI = comparative fit index; RMSEA = root-mean-square error of approximation; SRMR = standardized root-mean-square residual; M1, M2, M3 = Model 1, Model 2, Model 3. Difference test based on likelihood-ratio test, which generates corrected Δχ^2^ statistics when the maximum likelihood estimator MLR is used.

^a^Factor loadings over time freely estimated. ^b^ Factor loadings constrained to be equal over groups ^c^ Factor loadings and intercepts constrained to be equal over groups.
