# Supplementary material for: Development of coordination and muscular fitness in children and adolescents with parent-reported ADHD in the German longitudinal MoMo Study
Source: Sci Rep. 2022 Feb 8;12:2073. doi: 10.1038/s41598-022-06139-1 (PMC8827093; doi:10.1038/s41598-022-06139-1)
Supplement: Supplementary file 4 — Supplementary Table 2. [file 41598_2022_6139_MOESM4_ESM.docx]

**Supplementary Table 2**

*Invariance testing for coordination and muscular fitness over time (N =* *2,987)*

| Model | χ^2^ | | df | *p* | CFI | RMSEA |
| --- | --- | --- | --- | --- | --- | --- |
| **Coordination** | | | |  |  |  |
| M1: Configural invariance ^a^ | 29.89 | | 12 | 0.003 | 0.996 | 0.022 |
| M2: Metric invariance ^b^ | 32.33 | | 16 | 0.009 | 0.996 | 0.018 |
| M3: Scalar invariance ^c^ | 360.64 | | 20 | <0.001 | 0.925 | 0.076 |
| **M4: Partial scalar invariance ^d^** | **35.38** | | **18** | **0.009** | **0.996** | **0.018** |
| Difference between M1 and M2 | Δχ^2^ = 2.44, Δ*df =* 4, *p* = 0.655 | | | | | |
| Difference between M2 and M4 | Δχ^2^ = 3.05, Δ*df =* 2, *p* = 0.218 | | | | | |
| **Muscular fitness** | | | |  |  |  |
| M1: Configural invariance ^a^ | | 2.42 | 8 | 0.966 | 1 | 0 |
| M2: Metric invariance ^b^ | | 13.44 | 11 | 0.266 | 0.999 | 0.009 |
| M3a: Scalar invariance ^c^ | | 150.79 | 14 | <0.001 | 0.972 | 0.057 |
| **M3b: Partial scalar invariance ^d^** | | **20.89** | **12** | **0.052** | **0.998** | **0.016** |
| Difference between M1 and M2 | Δχ^2^ = 11.02, Δ*df =* 3, *p* = 0.012 | | | | | |
| Difference between M2 and M3b | Δχ^2^ = 7.45, Δ*df =* 1, *p* = 0.006 | | | | | |

*Note.* CFI = comparative fit index; RMSEA = root-mean-square error of approximation; SRMR = standardized root-mean-square residual; M1, M2, M3, M4 = Model 1, Model 2, Model 3, Model 4. Difference test based on likelihood-ratio test, which generates corrected Δχ^2^ statistics when the maximum likelihood estimator MLR is used.

^a^ Factor loadings over time freely estimated. ^b^ Factor loadings constrained to be equal over time ^c^ Factor loadings and intercepts constrained to be equal over time. ^d^ Factor loadings and intercepts constrained to be equal over time with the exception for one item.
